# Low 25-Hydroxyvitamin D Post-Kidney Transplant Is Associated with Increased Risk of BK Polyomavirus-Associated Nephropathy

**DOI:** 10.3390/microorganisms12122588

**Published:** 2024-12-13

**Authors:** Suseela A. Raj, Angela L. Zhou, Ekaterina Fedorova, Zhongyu Yuan, Didier A. Mandelbrot, Brad C. Astor, Sandesh Parajuli

**Affiliations:** 1 Division of Nephrology, Department of Medicine, University of Wisconsin School of Medicine and Public Health, Madison, WI 53705, USAzyuan94@wisc.edu (Z.Y.);; 2 Division of Transplantation, Department of Surgery, University of Wisconsin School of Medicine and Public Health, Madison, WI 53705, USA; 3 Department of Population Health Sciences, University of Wisconsin School of Medicine and Public Health, Madison, WI 53705, USA

**Keywords:** kidney transplants, vitamin D, BK virus

## Abstract

BK viremia (BKPyV-DNAemia) and nephropathy (BKPyVAN) are significant causes of morbidity and mortality in kidney transplant recipients (KTRs). Vitamin D supports immune function, yet low 25-hydroxyvitamin D [25(OH)D] is common among KTRs. The association between serum 25(OH)D, measured 61 days to 2 years post-transplant, and subsequent incident BKPyV-DNAemia and BKPyVAN was examined in KTRs without previous BKPyV-DNAemia or BKPyVAN, respectively. Out of 3308 KTRs, 399 (12%) were vitamin D deficient [25(OH)D ≤ 20 ng/mL], and 916 (27.7%) were insufficient [25(OH)D 21–29 ng/mL]. A total of 184 KTRs developed BKPyV-DNAemia and 44 developed BKPyVAN. The incidence rate (/100 person-years) for BKPyV-DNAemia was 2.88 in the 25(OH)D sufficient group, 2.22 in the insufficient group, and 2.37 in the deficient group. The incidence rate (/100 person-years) for BKPyVAN was 0.30 in the 25(OH)D sufficient group, 0.75 in the insufficient group, and 1.28 in the deficient group. Vitamin D deficiency (adjusted hazard ratio [aHR] compared to 25(OH)D sufficiency: 3.92; 95% CI: 1.66–9.23) and insufficiency (aHR: 2.22; 95% CI: 1.11–4.45) remained significantly associated with the incidence of BKPyVAN after adjustment for baseline characteristics. Low serum 25(OH)D was associated with an increased risk of BKPyVAN but not BKPyV-DNAemia.

## 1. Introduction

The BK polyomavirus is a common opportunistic infection after kidney transplantation and is clinically significant due to its ability to progress, causing increased morbidity and mortality [1,2,3]. Most of the general population is infected with BK virus in early childhood without significant disease [4,5,6]. After initial infection, BK virus can remain latent in the kidneys and uroepithelial cells [7,8]. In kidney transplant recipients (KTRs), the use of immunosuppressive agents to prevent graft rejection is associated with BK virus reactivation, replication, and progressive infection [2,9,10,11]. Infection is more common when KTRs are older or male, the donor had BK viruria, there is acute rejection, or with tacrolimus or high steroid exposure [12]. BK viruria is detected in about 30% of KTRs [13]. Infection with BK virus may progress from high-level viruria to BK viremia (BKPyV-DNAemia) in up to half of KTRs after 2–6 weeks, and, in a similar time frame, up to half of those with BKPyV-DNAemia may develop BK nephropathy (BKPyVAN), which is associated with increased total and death-censored graft failure [13,14,15]. Unfortunately, there are no effective antiviral therapies; treatment generally consists of decreasing immunosuppressive therapies [2,3,11].

Vitamin D plays an important role in immune function, with vitamin D deficiency and insufficiency associated with increased vulnerability to infection by various pathogens [16,17,18,19]. Vitamin D, in the form of active 1,25-dihydroxyvitamin D [1,25(OH)2-D], appears to promote the protective innate immune response and regulate the adaptive immune response [16,20,21]. Most immune cells, including T and B lymphocytes, monocytes, macrophages, and dendritic cells, have vitamin D receptors, through which vitamin D exerts effects, and immune cells can express CYP27B1 (1α-hydroxylase) to convert inactive 25-hydroxyvitamin D [25(OH)D] to active 1,25(OH)2-D [16,17,18,21]. The intracellular 1,25(OH)2-D produced by immune cells is key in regulating the immune response through genomic and non-genomic actions, such as autocrine and paracrine signaling [17,18]. Vitamin D’s effects on innate immunity include causing the production of antimicrobial products like cathelicidin and human beta-defensin from immune cells, enhancing the antimicrobial activity of monocytes and macrophages, and inhibiting dendritic cell maturation to promote more tolerogenic behavior [16,17,18,19,20,21,22]. Simultaneously, vitamin D regulates adaptive immunity by suppressing T and B lymphocyte activities [16,17,18,22]. Thus, vitamin D is associated with the overarching effect of decreasing infection via intracellular pathogen clearance [22].

The impact of vitamin D on immune function is relevant in the context of post-transplant infection. Inadequate levels of 25(OH)D are common among KTRs, with only about 15% of KTRs having adequate 25(OH)D around the time of transplantation [23,24]. Low 25(OH)D levels have been associated with increased post-transplant infection [25,26,27,28]. However, few studies have addressed BK virus specifically, and there is very little existing knowledge about the association of 25(OH)D level with BKPyV-DNAemia and BKPyVAN as distinct outcomes. The possible benefits of supplementation to correct vitamin D deficits are not fully defined for post-transplant outcomes [29].

In this study, we examined the association between serum 25(OH)D level and subsequent BKPyV-DNAemia and BKPyVAN in KTRs.

## 2. Materials and Methods

### 2.1. Study Population and Design

**Figure 1:**
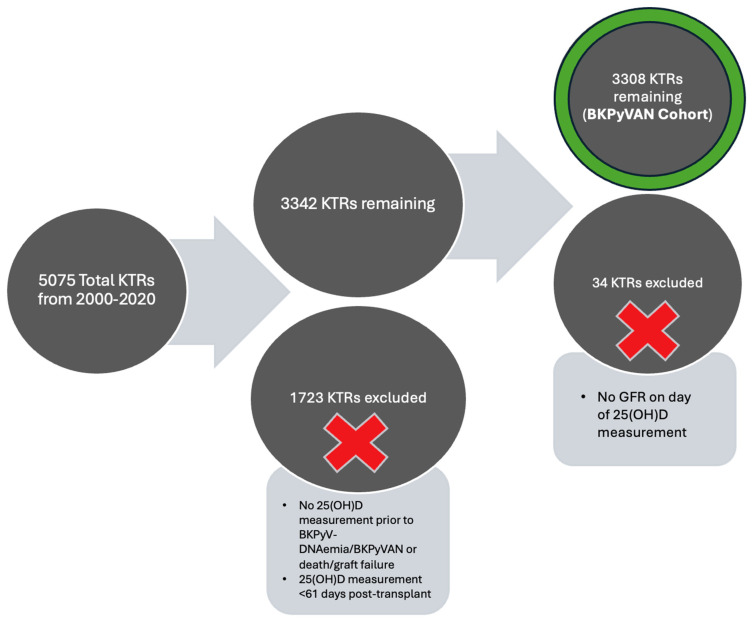
Process of inclusion and exclusion from analyses. 25(OH)D, 25-hydroxyvitamin D; BKPyV-DNAemia, BK viremia; BKPyVAN, BK nephropathy.

Study participants were drawn from the Wisconsin Allograft Recipient Database (WisARD). Recipients eligible for inclusion in the study were adults (≥18 years old) who received a kidney-only transplantation from 1 January 2000 to 31 December 2020, at the University of Wisconsin, survived with a functioning graft for a minimum of 6 months post-transplant, and had a serum 25(OH)D measurement available between 61 days and 2 years post-transplant. The first 25(OH)D level within this time period was used in analyses for KTRs with multiple 25(OH)D measurements available. Measurements of 25(OH)D performed ≤ 60 days post-transplant were not included due to increased variability in 25(OH)D levels, in addition to more frequent hospitalizations and acute events. This excluded a total of 1267 recipients from the study, while 3308 recipients remained included (Figure 1).

The primary outcomes of interest were incident BKPyV-DNAemia and BKPyVAN. Patients with BKPyV-DNAemia prior to the first eligible 25(OH)D measurement were excluded from analyses of BKPyV-DNAemia (BKPyV-DNAemia cohort) and those with BKPyVAN prior to the first eligible 25(OH)D measurement were excluded from analyses of BKPyVAN (BKPyVAN cohort) in order to increase the strength of any association found between 25(OH)D level and BKPyV-DNAemia or BKPyVAN. The University of Wisconsin Health Sciences Institutional Review Board approved this study.

### 2.2. Follow-Up

Recipients were followed from their first eligible serum 25(OH)D measurement, which was between 61 days and 2 years post-transplant, until the first instance of the outcome of interest or a maximum of 3 years. Recipients were censored at the time of graft failure or death.

### 2.3. Vitamin D and BK Virus Quantification

Serum 25(OH)D measurements were performed at the University of Wisconsin Hospital and Clinics Clinical Laboratory and other qualified laboratories with liquid chromatography-tandem mass spectrometry. Vitamin D categories were defined based on the Endocrine Society criteria, with vitamin D insufficiency being 25(OH)D of 21–29 ng/mL and deficiency being 25(OH)D ≤ 20 ng/mL [30]. Serum 25(OH)D was monitored within one year post-transplant and then annually for most KTRs. All recipients were discharged after transplant on calcium and cholecalciferol 1000 units po twice daily and were instructed to follow a vitamin D-rich diet.

BKPyV-DNAemia was defined as >1000 copies/mL of BK viral load in plasma on polymerase chain reaction (PCR) within 2 years post-transplant. This definition was based on the cut-off that designates clinically significant BKPyV-DNAemia requiring management at our institution and agrees with guideline-endorsed practice [14]. All samples were analyzed at our institution, whose BK PCR assay has an analytic measurement range of 250–10,000,000 copies/mL of plasma and a detection range of 50 to unlimited copies/mL of plasma. BKPyVAN was defined as positive simian virus 40 staining on renal biopsy.

### 2.4. BK Monitoring and Treatment Protocols

At our institution, quantitative plasma BK PCR is measured every 2 weeks for the first 3 months post-transplant, monthly from 3–12 months, and at the time of a renal allograft biopsy. If serum BK PCR is detectable, KTRs are monitored every 2 weeks until PCR is negative for three consecutive measurements. In the case of treatment for allograft rejection, BK PCR is monitored every 2 weeks [31].

Patients with BKPyV-DNAemia underwent renal allograft biopsy if there was a rise in serum creatinine >25% from the baseline [32]. Recipients with stable graft function and BKPyV-DNAemia did not undergo biopsy. Biopsies at our institution are also performed in the setting of an unexplained rise in serum creatinine, proteinuria, or de novo donor-specific antibodies, or in patients with pre-transplant donor-specific antibodies according to protocol at 3 and 12 months post-transplant [1].

BK infection management at our institution parallels current guidelines, centering on protocolized immunosuppressive adjustments based on plasma BK PCR level [14]. For BK PCRs < 1000 copies/mL, PCR is monitored but immunosuppressives are not adjusted. For BK PCR 1000–10,000 copies/mL, the antimetabolite is decreased by 25%, and for BK PCRs > 10,000 copies/mL, the antimetabolite is decreased by 50%. If BK PCR remains >10,000 copies/mL after 2 weeks following regimen changes, immunosuppressives continue to be reduced aggressively, including decreases in calcineurin inhibitor (CNI) targets. Adjustment of CNI dose may be considered before antimetabolite adjustment per physician discretion. Adjunctive agents are used per physician discretion, such as intravenous immunoglobulin (IVIG), leflunomide, and cidofovir. BKPyVAN is managed with immunosuppressive reduction and adjunctive agents, primarily IVIG [1].

### 2.5. Other Characteristics

At the time of kidney transplantation, information about demographics, body mass index (BMI), cause of end-stage kidney disease (ESKD), living donor, prior transplantation, delayed graft function, immunosuppressive medications, preemptive transplantation prior to initiation of dialysis, quantity of human leukocyte antigen mismatches, and CMV serostatus was collected. CMV serostatus was considered high-risk when Donor+/Recipient-. BMI was defined by weight/height^2^ (kg/m^2^). Delayed graft function was interpreted as requiring hemodialysis within the first week post-transplant. The number of days post-transplant at the first serum 25(OH)D measurement was also recorded. The estimated glomerular filtration rate (eGFR) was calculated from a serum creatinine measurement taken on the same day as the first serum 25(OH)D measurement, and KTRs with an eGFR < 15 mL/min/1.73 m^2^ were not included. History of acute rejection before the first 25(OH)D measurement was also recorded and was identified based on a clinical renal pathologist’s diagnosis from renal biopsy and the Banff Criteria [33].

### 2.6. Statistical Analysis

Linear regression models or chi-square tests were used to compare baseline characteristics across 25(OH)D categories, assessed at the first applicable 25(OH)D measurement. Incidence rates were compared between 25(OH)D categories using Kaplan–Meier curves and log-rank tests. Cox proportional hazards models were used to assess the independent association of 25(OH)D categories with BKPyV-DNAemia and BKPyVAN. Multivariable restricted cubic spline Poisson regression models with knots at the 10th, 50th, and 90th percentiles were utilized to assess continuous associations of 25(OH)D level with events. Analyses were carried out with Stata Statistical Software Release 13.1 (www.stata.com accessed on 23 February 2024).

## 3. Results

### 3.1. Baseline Characteristics

Of the 3308 KTRs without BKPyVAN prior to the first eligible 25(OH)D measurement, 399 (12%) were vitamin D deficient [25(OH)D ≤ 20 ng/mL], and 916 (27.7%) were insufficient [25(OH)D 21–29 ng/mL] (Table 1). Compared to the vitamin D sufficient group, recipients in the vitamin D insufficient and deficient groups were 1.7 and 5.1 years younger on average, respectively. The vitamin D insufficient and deficient groups had a mean BMI 0.6 kg/m^2^ and 0.3 kg/m^2^ higher, respectively, than that of the sufficient group. Additionally, the insufficient group was 24.2% of non-white race and the deficient group was 28.1% of non-white race, compared to just 18.0% non-white in the sufficient group. Furthermore, the mean eGFR was 73.7 in both the insufficient and deficient groups, compared to a lower eGFR of 69.9 in the sufficient group. Recipients with lower 25(OH)D also were more likely to have ESKD due to diabetes mellitus, have a non-living donor, have had prior transplantation, have a history of acute rejection, have delayed graft function, have received thymoglobulin and alemtuzumab for induction immunosuppression, not be on tacrolimus-based immunosuppression, and not have had preemptive transplantation. Results were largely similar for those without BKPyV-DNAemia prior to the first eligible 25(OH)D measurement.

### 3.2. Incidence of BK Viremia

**Figure 2:**
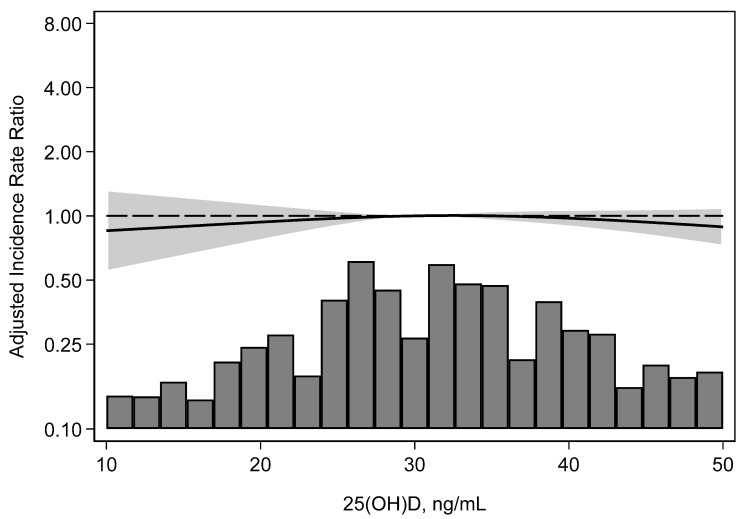
Adjusted incidence rate ratio for BKPyV-DNAemia, by 25(OH)D level. No significant association of BKPyV-DNAemia with 25(OH)D levels. 25(OH)D, 25-hydroxyvitamin D; BKPyV-DNAemia, BK viremia.

In total, 184 KTRs developed BKPyV-DNAemia. Serum 25(OH)D level was not associated with any significant pattern of BKPyV-DNAemia incidence (Table 2). The incidence rate of BKPyV-DNAemia was 2.88 per 100 person-years in the 25(OH)D sufficient group, 2.22 in the insufficient group, and 2.37 in the deficient group. The incidence rate of BKPyV-DNAemia did not differ significantly between those with sufficient or insufficient 25(OH)D (*p* = 0.22). The continuous association of 25(OH)D with BKPyV-DNAemia incidence across all 25(OH)D levels revealed no significant association (Figure 2).

### 3.3. Incidence of BK Nephropathy

**Figure 3:**
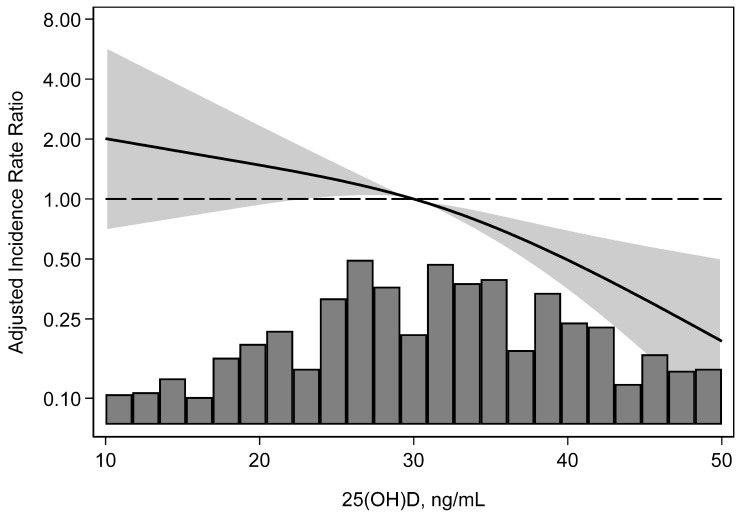
Adjusted incidence rate ratio for BKPyVAN, by 25(OH)D level. The incidence of BKPyVAN was higher with a lower 25(OH)D level. 25(OH)D, 25-hydroxyvitamin D; BKPyVAN, BK nephropathy.

In total, 44 KTRs developed BKPyVAN. The incidence rate for BKPyVAN/100 person-years was 0.30 in the 25(OH)D sufficient group, 0.75 in the insufficient group, and 1.28 in the deficient group (Table 3). Recipients who were 25(OH)D deficient had a 4.15-fold higher incidence of BKPyVAN (relative hazard [RH] = 4.15; 95% CI, 1.94–8.86), and those who were 25(OH)D insufficient had a 2.41-fold higher incidence of BKPyVAN (RH = 2.41; 95% CI, 1.20–4.83) compared to those who were 25(OH)D sufficient. Adjustment for multiple baseline characteristics gave similar results, with 25(OH)D deficiency associated with a 3.92-fold higher incidence (adjusted hazard ratio [aHR] = 3.92; 95% CI, 1.66–9.23) and insufficiency associated with a 2.22-fold higher incidence (aHR = 2.22; 95% CI, 1.11–4.45) of BKPyVAN compared with 25(OH)D sufficient KTRs. The continuous association of 25(OH)D with BKPyVAN incidence across all 25(OH)D levels revealed that the incidence of BKPyVAN was monotonically higher with lower serum 25(OH)D level (Figure 3).

## 4. Discussion

This study examined the association of serum 25(OH)D level with an incidence of BKPyV-DNAemia and BKPyVAN in kidney transplant recipients. We hypothesized that low 25(OH)D is associated with a greater risk of BK virus progression, given that vitamin D has a known role in immune function and low 25(OH)D has been associated with increased post-transplant infection [16,17,25,26,27,28]. We found that nearly 40% of KTRs were either 25(OH)D deficient or insufficient, according to serum measurement between 61 days and 2 years post-transplant (Table 1). Risk factors for lower 25(OH)D included younger age, non-white race, diabetes, prior transplantation, no preemptive transplantation, history of acute rejection, and higher eGFR, among other factors. These demographics largely mirrored those noted in a study carried out at our institution by Astor et al. [25], apart from our findings on eGFR and preemptive transplantation. Furthermore, results revealed that 25(OH)D deficiency and insufficiency are associated with a higher incidence of BKPyVAN, but a low 25(OH)D level is not associated with a higher incidence of BKPyV-DNAemia.

The immune system is responsible for clearing infection. Following kidney transplantation, immunosuppressive regimens prevent graft rejection but also put KTRs at increased risk of infection by opportunistic pathogens, like BK virus [2,9,10,11]. Vitamin D is important in proper immune function, as most immune cells have vitamin D receptors and express 1α-hydroxylase to convert vitamin D to its active form [16,17,18,21]. Vitamin D increases the antimicrobial effects of the innate immune system while regulating T and B lymphocyte actions in the adaptive immune system [16,17,18,19,20,21,22]. Overall, vitamin D is associated with increased intracellular pathogen clearance, which is essential during infections with BK virus and other pathogens [22]. Based on their study of KTRs who received calcitriol supplementation pre- and post-transplant, Moscarelli et al. [34] suggest that sufficient vitamin D, measured as 1,25(OH)2-D3, supports a more efficient humoral response to CMV and BK virus infection by increasing immunoglobulin, γ-globulin, and CD19+ B cell concentrations in KTRs, even with concomitant therapy with immunosuppressant mycophenolic acid.

Opportunistic infections are common in KTRs and can have detrimental effects, yet research associating vitamin D with post-transplant opportunistic infections is not fully conclusive, with few studies addressing BK virus specifically, as we have done in our study. Furthermore, our study is unique in that we have taken data from a large sample size, over 3000 kidney transplant recipients over a 20-year span, and we have analyzed BKPyV-DNAemia and BKPyVAN as two separate outcomes of interest.

The previous literature has demonstrated vitamin D deficiency is a risk factor for CMV infection in KTRs [25,35,36]. In a group of 1976 recipients who received kidney transplantations at our institution, Astor et al. [25] found vitamin D deficiency [25(OH)D ≤ 20 ng/mL] was associated with a 1.81-fold higher risk of late cytomegalovirus (CMV) infection than vitamin D sufficiency, after adjustment for various characteristics. Low vitamin D levels have also been associated with increased post-transplant opportunistic infections in general, with vitamin D sufficiency lowering the chance of infection [26,27,28,34]. In a prospective observational cohort study of 246 KTRs, Fernández-Ruiz et al. [27] found vitamin D deficiency, defined more narrowly [25(OH)D < 12 ng/mL], at 1 month post-transplant was a risk factor for overall and, more notably, opportunistic infection, with a dose–response gradient across decreasing quartiles of 25(OH)D. Rech et al. [26] observed, in a retrospective review of 89 recipients, that vitamin D insufficiency [25(OH)D ≤ 20 ng/mL] within 30 days of kidney transplantation was associated with increased incidence of CMV and BK virus infection during the first year post-transplant, and BK virus infection, defined as biopsy-proven or detectable by PCR, occurred less frequently in vitamin D sufficient than insufficient patients. The small cohort of this study was a disadvantage, and, unlike our study, this study did not differentiate BKPyV-DNAemia and BKPyVAN as separate outcomes. Also in support of an association between vitamin D level and opportunistic infection, Moscarelli et al. [34] observed in a retrospective study of 373 recipients that 1,25(OH)2-D3 deficiency (<20 pg/mL) measured post-transplant was a risk factor for CMV infection and BK virus infection, defined as blood viral load >1000 copies/mL using PCR.

Our study suggests sufficient 25(OH)D post-transplant is associated with lower incidence of BKPyVAN in KTRs. However, the best method to obtain sufficient vitamin D levels to mitigate opportunistic infection remains unclear. Moscarelli et al. [34] found administration of calcitriol for at least 1 month pre-transplantation and during follow-up allowed obtainment of adequate 1,25(OH)2-D3 level, and recipients using calcitriol had a lower incidence of BK viremia, at 7% versus 19% of control recipients, and polyomavirus-associated nephropathy (PVAN), with only one user developing PVAN compared to 15 controls. However, Rech et al. [26] observed that recipients who received vitamin D supplementation, ergocalciferol, or cholecalciferol, after transplantation, showed no difference in the frequency of opportunistic infection compared to those who did not take supplements. Additionally, because those with chronic kidney disease have decreased renal parenchyma, leading to less 1α-hydroxylase to convert 25(OH)D to active 1,25(OH)2-D3, KTRs may require both an active vitamin D analog and a source of vitamin D2 or D3 to maintain 25(OH)D levels for use in extrarenal tissues, like immune cells [37]. Thus, supplementation before transplantation may be more effective in modifying the risk of opportunistic infection, but further studies are needed to determine the most advantageous timing and form of supplementation.

The limitations of our study include potential selection bias, as KTRs were included based on the timing of 25(OH)D measurements performed for clinical purposes. In addition, the study followed an observational design, which prevented controlling for all possible confounding variables, but we attempted to mitigate potential residual confounding variables by adjusting for multiple baseline characteristics. In addition, we were not able to include other metrics relevant to vitamin D levels, such as 1,25(OH)2-D3, serum calcium, phosphorus, parathyroid hormone, etc. We also did not account for variations in dietary intake of vitamin D or vitamin D supplementation among recipients and its potential impact on the outcomes of interest. Furthermore, 25(OH)D was based on the first measurement within 61 days to 2 years post-transplant, and subsequent changes in 25(OH)D level after this initial measurement were not accounted for. Additionally, this was a single-center study, and our findings from KTRs largely from Wisconsin and the Midwest may not translate to other locations with greater ultraviolet B exposure and less prevalent 25(OH)D deficiency. We also did not account for fluctuations in 25(OH)D level due to seasonal ultraviolet B exposure changes, which is a limitation given that recipients had 25(OH)D levels measured during different seasons throughout the year.

In summary, this study found low serum 25(OH)D after kidney transplantation to be associated with an increased risk of developing BKPyVAN but not BKPyV-DNAemia. Furthermore, certain characteristics in KTRs predicted low 25(OH)D. These findings reinforce the role of vitamin D in immune function and pathogen clearance, underscoring the importance of further research on the impact of vitamin D levels in transplant recipients and more intensive clinical monitoring of vitamin D levels, especially in certain recipients. Furthermore, these findings highlight the need for more clinical trials to evaluate the potential benefits of vitamin D supplementation in regulating vitamin D levels and mitigating complications associated with BKPyVAN and other post-transplant infections.

## Figures and Tables

**Table 1:** Baseline characteristics, by 25(OH)D category, based on the BKPyVAN cohort.

		Overall (N = 3308)	Serum 25(OH)D			
			≥30 ng/mL (N = 1993)	21–29 ng/mL (N = 916)	≤20 ng/mL (N = 399)	*p*
Characteristics at time of transplant						
Age, years (SD)		52.6 (12.9)	53.7 (12.4)	52.0 (13.2)	48.6 (13.5)	<0.001
Female, %		39.8	38.4	42.0	41.1	0.11
Non-white, %		20.9	18.0	24.2	28.1	<0.001
Cause of ESKD, %						
	Diabetes mellitus	25.0	21.0	29.9	33.6	<0.001
	Hypertension	12.3	12.9	11.2	12.3	
	PKD	14.1	17.4	9.5	8.8	
	Glomerulonephritis	25.0	26.2	24.0	21.1	
	Other	23.6	22.6	25.3	24.3	
Body mass index, kg/m^2^ (SD)		28.1 (5.3)	27.9 (5.2)	28.5 (5.3)	28.2 (5.4)	0.04
Living donor, %		38.9	42.4	34.9	30.3	<0.001
Prior transplant, %		20.7	38.9	41.4	45.4	<0.001
Delayed graft function, %		18.3	36.0	40.6	44.5	<0.001
Induction immunosuppression						
	Thymoglobulin	31.6	30.0	33.1	36.6	0.003
	Basiliximab	55.6	57.1	55.8	47.9	
	Alemtuzumab	11.1	10.8	10.3	14.0	
	Other	1.7	2.1	0.9	1.5	
Preemptive transplantation		23.5	29.3	16.4	10.8	<0.001
HLA mismatch, %						
	0–2	19.4	19.6	18.7	19.8	0.90
	3–4	43.8	43.0	44.4	41.9	
	5–6	36.8	36.5	36.9	38.4	
High-risk cytomegalovirus serostatus, %		11.1	30.6	32.9	32.6	0.16
Tacrolimus-based immunosuppression		92.0	93.7	90.8	86.2	<0.001
History of acute rejection before the first serum 25(OH)D measurement, %		10.3	9.2	8.8	19.5	<0.001
eGFR at the time of the first serum 25(OH)D measurement (SD)		71.4 (25.2)	69.9 (24.1)	73.7 (26.6)	73.7 (27.0)	<0.001
Time of first serum 25(OH)D measurement, number of days post-transplant (SD)		247 (141)	247 (133)	240 (147)	263 (161)	0.24

**Table 2:** Incidence of BKPyV-DNAemia, by 25(OH)D category.

	Serum 25(OH)D			
	≥30 ng/mL	21–29 ng/mL	≤20 ng/mL	P-Trend
Numbers of events	120	44	20	-
Incidence rate/100 person-years	2.88	2.22	2.37	-
Relative hazard (95% confidence interval)	1.0 Reference	0.70 (0.50, 0.99) *p* = 0.05	0.80 (0.50, 1.28) *p* = 0.35	0.10
Adjusted * relative hazard (95% confidence interval)	1.0 Reference	0.72 (0.50, 1.02) *p* = 0.07	0.88 (0.55, 1.42) *p* = 0.61	0.22

* Adjusted for age, sex, race, cause of end stage kidney disease, body mass index, donor status, prior transplant, delayed graft function, induction immunosuppression, HLA mismatch category, cytomegalovirus serostatus, history of acute rejection, estimated glomerular filtration rate category, and time since transplantation. 25(OH)D, 25-hydroxyvitamin D; BKPyV-DNAemia, BK viremia.

**Table 3:** Incidence of BKPyVAN, by 25(OH)D category.

	Serum 25(OH)D			
	≥30 ng/mL	21–29 ng/mL	≤20 ng/mL	P-Trend
Numbers of events	15	17	12	-
Incidence rate/100 person-years	0.30	0.75	1.28	-
Relative hazard (95% confidence interval)	1.0 Reference	2.41 (1.20, 4.83) *p* = 0.01	4.15 (1.94, 8.86) *p* < 0.001	<0.001
Adjusted * relative hazard (95% confidence interval)	1.0 Reference	2.22 (1.11, 4.45) *p* = 0.02	3.92 (1.66, 9.23) *p* = 0.002	0.001

* Adjusted for age, sex, race, cause of end stage kidney disease, body mass index, donor status, prior transplant, delayed graft function, induction immunosuppression, HLA mismatch category, cytomegalovirus serostatus, history of acute rejection, estimated glomerular filtration rate category, and time since transplantation. 25(OH)D, 25-hydroxyvitamin D; BKPyVAN, BK nephropathy.

## Data Availability

The data that support the findings of this study are available from the corresponding author [SP], upon reasonable request.

## Abbreviations

1,25(OH)2-D	1,25-dihydroxyvitamin D
25(OH)D	25-hydroxyvitamin D
aHR	adjusted hazard ratio
BKPyV-DNAemia	BK viremia
BKPyVAN	BK polyomavirus-associated nephropathy
BMI	body mass index
CMV	cytomegalovirus
CNI	calcineurin inhibitor
eGFR	estimated glomerular filtration rate
ESKD	end-stage kidney disease
IVIG	intravenous immunoglobulin
KTRs	kidney transplant recipients
PCR	polymerase chain reaction
PVAN	polyomavirus-associated nephropathy
RH	relative hazard
SD	standard deviation
WisARD	Wisconsin Allograft Recipient Database
